# Residential proximity to major roads and adverse birth outcomes: a hospital-based study

**DOI:** 10.1186/1476-069X-12-34

**Published:** 2013-04-18

**Authors:** Takashi Yorifuji, Hiroo Naruse, Saori Kashima, Soshi Takao, Takeshi Murakoshi, Hiroyuki Doi, Ichiro Kawachi

**Affiliations:** 1 Department of Human Ecology, Okayama University Graduate School of Environmental and Life Science, 1-1-1 Tsushima-naka, Kita-ku, Okayama 700-8530, Japan; 2 Department of Obstetrics, Seirei Hamamatsu General Hospital, 2-12-12 Sumiyoshi, Naka-ku, Hamamatsu, Shizuoka 430-8558, Japan; 3 Department of Public Health and Health Policy, Institute of Biomedical & Health Sciences, Hiroshima University, 1-2-3 Kasumi, Minami-ku, Hiroshima 734-8551, Japan; 4 Department of Epidemiology, Okayama University Graduate School of Medicine, Dentistry and Pharmaceutical Sciences, 2-5-1 Shikata-cho, Kita-ku, Okayama 700-8558, Japan; 5 Department of Society, Human Development, and Health, Harvard School of Public Health, 677 Huntington Ave, Boston, MA 02115, USA

**Keywords:** Air pollution, Diabetes mellitus, Geographic Information System, Hypertension, Low birth weight, Pregnancy outcomes, Preterm birth, Socio-economic position, Smoking

## Abstract

**Background:**

Exposure to air pollution has been demonstrated to increase the risk of preterm birth and low birth weight (LBW). Although evidence has accumulated on characteristics associated with increased risk of air pollution-related health effects, most studies have been conducted in the adult population and evidence on reproductive outcomes is limited. We examined whether socio-economic position (SEP) and parental characteristics (parental behavior and co-morbidity) modified the relationship between air pollution and adverse birth outcomes.

**Methods:**

Data were extracted from a perinatal hospital database based in Shizuoka, Japan. We restricted the analysis to mothers who delivered live-born single births from January 1997 to December 2010 (n = 16,615). Each birth was assigned proximity to major roads. Multivariate adjusted odds ratios (ORs) and their 95% confidence intervals (CIs) were estimated for the outcomes of preterm birth and term LBW. We stratified subjects by individual/area-level SEP and parental characteristics. We then measured interactions on the additive scale between the respective factors and exposure.

**Results:**

Lower SEP at both individual and area levels was associated with the increased occurrence of adverse birth outcomes. Living within 200 m from a major road increased the risk of preterm birth by 1.5 times (95% CI: 1.3-1.9) and LBW by 1.2 times (95% CI: 0.9-1.6). Mothers with lower individual SEP defined by household occupation experienced higher ORs for term LBW (OR = 3.1, 95% CI: 1.2-8.2) compared with those with higher individual SEP. In contrast, mothers who lived in the highest area-level SEP region (i.e., affluent areas) showed slightly higher point estimates compared with those who lived in middle or poor areas. In addition, maternal diabetic and hypertensive status modified the association between proximity and preterm birth, while maternal smoking status modified the association between proximity and term LBW.

**Conclusions:**

The present study demonstrated that air pollution is an independent risk factor for adverse birth outcomes. Mothers with lower individual SEP and mothers living in higher SEP region may be susceptible to the adverse effect of air pollution. Maternal diabetic, hypertensive, and smoking status may also increase susceptibility to this air pollution-related health effect.

## Background

Evidence has accumulated on the association between air pollution and adverse birth outcomes, such as preterm birth or low birth weight (LBW) [1-4]. Indeed, recent systematic reviews and original studies have indicated positive associations between various air pollution indicators (e.g., gaseous pollutants, particulate matter, and air pollution surrogates) and birth outcomes [5-8].

Recently, several studies have attempted to identify characteristics associated with increased risk of air pollution-related health effects, e.g. age, preexisting cardiovascular disease, diabetes, and low socio-economic position (SEP) [9,10]. However, most studies were conducted in the adult population, and evidence concerning reproductive outcomes is limited and potentially conflicting [11].

Because SEP is a robust predictor of health outcomes, it has been questioned whether SEP is a confounder or an effect modifier of the association between air pollution and adverse health outcomes [12]. A growing number of studies have therefore sought to examine the simultaneous impacts of SEP and air pollution, although most studies considered SEP a confounder. Evidence concerning group/area-level SEP modification is somewhat conflicting [13-17], but many studies have observed effect modification by individual SEP, i.e. lower SEP individuals may be more susceptible to adverse effects of air pollution, which may in turn be related to underlying vulnerability (co-morbidity or adverse health behavior such as smoking) [18-21].

In the field of air pollution and reproductive epidemiology, four studies have examined how SEP potentially modified the relationships between air pollution and adverse birth outcomes [22-25]. Three of these used a distance-based exposure index as the air pollution indicator, but reported potentially conflicting results. Two studies in California, USA, observed increased effect estimates for preterm birth and LBW in low SEP census areas [23,24], while a study in Canada demonstrated the opposite result, i.e. increased pollution-related effects for preterm birth, LBW, and small-for-gestational-age birth in subjects who had higher SEP both at the individual and group/district level [22]. Another study in Korea, which used particulate matter concentration (PM_10_) as the exposure index, found a higher effect estimate for preterm birth in low SEP regions [25].

Moreover, few studies have evaluated how parental characteristics (except SEP characteristics) modify the association between air pollution and reproductive outcomes, although the findings may provide important insights for possible mechanisms [3]. One study in New Jersey, USA, observed increased effect estimates for small-for-gestational-age birth among pregnant women with pregnancy complications (i.e. placental abruption and premature rupture of the membrane) [26].

In the present study, we therefore used a hospital-based perinatal database to evaluate how SEP at both individual and group/area levels and parental characteristics potentially modify the relationship between air pollution and adverse birth outcomes (preterm birth and term LBW).

## Methods

### Participants

**Figure 1:**
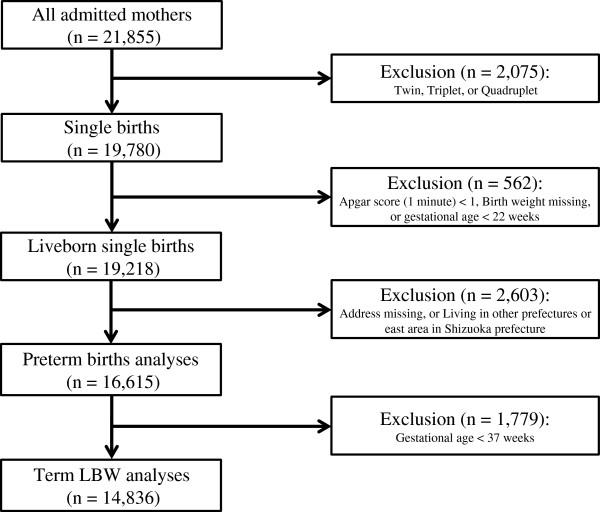
**Selection of eligible births.** When term low birth weight (LBW), i.e., birth weight <2500 g, was adopted as the health outcome, we restricted analyses to mothers who delivered term births (born at 37 or more completed weeks of gestation).

Data were extracted from the perinatal database maintained at the Seirei Hamamatsu General Hospital, Shizuoka, Japan since January 1997. The hospital is the main perinatal medical center in the western part of Shizuoka prefecture (west of Tokyo). The hospital reported 1582 new live births in 2010, and approximately one-eighth of the babies born in the western part of Shizuoka were born in this hospital [8]. The database contains information from all mothers admitted to the department of obstetrics in the hospital (n = 21,855 from 1997 to 2010). In this study, we restricted analysis to live-born single births delivered from January 1997 to December 2010. We assumed that babies with an Apgar score of >1 one minute after birth were live-born births. We thus defined the eligibility criteria for inclusion in this study as follows: single births, babies whose Apgar score was >1 one minute after birth, babies who had birth weight data, and deliveries after 22 weeks gestational age. We retrieved 19,218 births from the database (Figure 1). Details of the methodology used in the current study have been published elsewhere [8,27].

The availability of residential information at delivery was a prerequisite for inclusion in the study because we used proximity to major roads as an index for air pollution exposure; as a result of this criterion we excluded 206 births. Furthermore, since the hospital is located in the western part of Shizuoka prefecture, we restricted births to those whose mothers lived in the western part of Shizuoka prefecture, because mothers who lived in central or eastern parts were likely to have returned to their parents’ home during pregnancy to give birth (a Japanese tradition). However, we could not obtain information regarding maternal movement. When we adopted term LBW as a health outcome, we further restricted the analysis to mothers who delivered term births (born at 37 or more weeks of gestation). Our final set of participants included 16,615 births for preterm birth and 14,836 births for term LBW analyses (Figure 1).

### Exposure data

We used mothers’ residential proximity to major roads, which was defined according to the amount of traffic, as an index for air pollution exposure. First, we identified the exact residential address at delivery for each birth and measured proximity to major roads for all available mothers’ addresses. We categorized proximity according to the following groups (≤200 m; >200 m) based on previous epidemiological studies [8,27-29] and exposure assessment studies showing exponential decay in exposure with increasing distance from major roads [30,31]. We also employed an alternative trichotomized exposure indictor (≤50, 50–200, >200 m) to examine a possible dose–response relationship between proximity to major roads and adverse outcomes.

Major roads were defined as those having total vehicle counts greater than 50,000 per 24 hours on a weekday [32,33]. Road type and traffic volume data were obtained from the 2005 Road Traffic Census conducted by the Road Bureau of the Ministry of Land, Infrastructure, Transport, and Tourism. The major roads defined in this study corresponded almost exactly with the existing expressways or primary national highways. All geographic variables were collected by the Geographic Information System (GIS) software ArcGIS (ESRI Japan Inc., version 9.3).

### Outcome data

We examined two adverse birth outcomes: preterm birth and term LBW. Preterm births were defined as births less than 37 weeks gestational age. Gestational ages were measured based on the last menstrual period, and mostly confirmed or corrected by ultrasound measurements at approximately 10 weeks gestational age. Term LBW was defined as birth weight <2500 g among term newborns.

### Individual/area-level SEP and personal characteristics

We retrieved information about individual characteristics from the perinatal database. Individual information was obtained from mothers by trained obstetricians or midwives at the time of prenatal checkup when the expected delivery date was confirmed, and added or corrected during admission. Occupation of the mother or father (whichever was higher) was used as our measure of individual SEP. Although occupation was available in five categories (professional workers; employees; self-employed workers; part-time workers; unemployed and not active in the labor force including housewives), we combined the last three categories because of limited numbers in the last two categories.

We also extracted the following personal characteristics: maternal age, maternal smoking (never smoked; ex-smoker including mothers who quit smoking during pregnancy; current-smoker), maternal alcohol consumption (drinker; non-drinker), paternal smoking (never smoked; ex- or current-smoker), maternal obesity (before pregnancy), maternal past history of diabetes, and maternal past history of hypertension. Maternal obesity (before pregnancy) was defined as mothers whose body mass index (BMI) was more than 25. BMI was calculated as body weight before pregnancy (kg) divided by height squared (m^2^).

Area-level SEP (at the district level) was measured as the proportion of professional workers and the proportion of production or transportation workers over 15 years of age in the corresponding census region where the mothers lived. Data were obtained from the 2000 national census. The study area included 1872 census regions. In the analyses examining effect modification by area-level SEP, the SEP level was tertiled as previous studies used trichotomized categories of regional SEP [23,25].

### Data analyses

First, we calculated the proportions of preterm births and term LBW according to air pollution exposure category (≤200 m; >200 m), personal characteristics, and individual/area-level SEP. We then examined the distribution of individual/area-level SEP and personal characteristics separated by proximity to major roads.

We next estimated the multivariate adjusted odds ratios (ORs) for preterm birth or term LBW according to major roads using a logistic regression model. We first obtained crude ORs (Model 1) and then adjusted for maternal age, newborn’s sex, and personal characteristics that could be considered as potential confounders (Model 2) as follows: household occupation (professionals; employees; self-employed/part-time workers/unemployed), maternal smoking (never smoked; ex-smoker; current-smoker), maternal alcohol consumption (drinker; non-drinker), paternal smoking (ex- or current-smoker; non-smoker), and maternal BMI. We further adjusted for individual SEP (Model 3). Finally, we additionally adjusted for area-level SEP (Model 4), i.e. the proportion of professional workers in the corresponding census. Gestational age was also included in all models in the analyses for LBW. Maternal BMI, the proportion of professional workers, and gestational age were treated as continuous variables. Maternal age was entered as a linear and quadratic term into the models because a U-shaped association was expected between maternal age and adverse birth outcomes.

We also employed the alternative trichotomized exposure indictor (≤50, 50–200, >200 m) instead of the simple dichotomization (≤200 m; >200 m) and repeated the analyses.

To examine effect modification by individual/area-level SEP and parental characteristics, we stratified the subjects by individual/area-level SEP and parental characteristics and applied the final fully-adjusted logistic models (i.e., maternal age, newborn’s sex, personal characteristics, and individual/area-level SEP). Each variable was excluded when the subjects were stratified by the corresponding variable. We then measured interactions on the additive scale between the respective factors and exposure. We used Excel (Microsoft Corporation, Redmond, WA, USA) spreadsheets provided by Knol and VanderWeele [34] and calculated the proportion attributable to the interaction (AP), the proportion of disease among those with both exposures that is attributable to their interaction [35]. In the absence of interaction, AP equals 0. AP > 0 means positive interaction or more than additivity and AP < 0 means negative interaction or less than additivity.

All confidence intervals (CIs) were estimated at the 95% level. The PASW software package (Version 18.0 J, SPSS Japan Inc., Tokyo, Japan) was used for statistical analyses. Approval for this study was obtained from the Institutional Review Board of Seirei Hamamatsu General Hospital and Okayama University (No 498).

## Results

Proportions of preterm births and term LBW according to demographic characteristics are shown in Table 1. Preterm births and LBW were observed more often in areas closer to major roads. Additionally, younger and older mothers tended to experience more adverse birth outcomes, especially for term LBW. Regarding SEP variables, as expected, mothers with higher individual SEP (defined by household occupation) and mothers who lived in higher area level SEP regions tended to have fewer preterm births. Furthermore, while maternal smoking was associated with both outcomes, maternal obesity was associated with an increased risk of preterm birth, but decreased risk of term LBW.

**Table 1:** Proportions of preterm births and term low birth weight by characteristics of parents, newborns, and census region

	**Births**	**PTB**	**Term births**	**Term LBW**
	**n = 16,615**	**n (%)†**	**n = 14,836**	**n (%)†**
**Parental variables**				
Proximity to major roads				
≤200 m	953	149 (15.6)	804	80 (10.0)
>200 m	15662	1630 (10.4)	14032	1145 (8.2)
Maternal age				
<20	2096	236 (11.3)	1860	183 (9.8)
20-34	11663	1216 (10.4)	10447	824 (7.9)
≥35	2854	327 (11.5)	2527	218 (8.6)
Household occupation				
Professional workers	1854	143 (7.7)	1711	121 (7.1)
Employees	13070	1408 (10.8)	11662	995 (8.5)
Self-employed/Part-time workers/Unemployed	952	106 (11.1)	846	63 (7.4)
Maternal smoking				
Never smoked	15545	1607 (10.3)	13938	1129 (8.1)
Ex- or current-smoker	877	145 (16.5)	732	82 (11.2)
Maternal alcohol consumption				
Non-drinker	15674	1672 (10.7)	14002	1159 (8.3)
Drinker	757	82 (10.8)	675	53 (7.9)
Paternal smoking				
Never smoked	8650	874 (10.1)	7776	620 (8.0)
Ex or current-smoker	7152	774 (10.8)	6378	545 (8.5)
Maternal obesity				
Not obese	15215	1574 (10.3)	13641	1149 (8.4)
Obese	1348	183 (13.6)	1165	72 (6.2)
Maternal diabetes				
No diabetes	15983	1704 (10.7)	14279	1180 (8.3)
Diabetes	617	72 (11.7)	545	44 (8.1)
Maternal hypertension				
No hypertension	15707	1672 (10.6)	14035	1149 (8.2)
Hypertension	893	104 (11.6)	789	74 (9.4)
**Newborn variables**				
Sex				
Male	8571	1006 (11.7)	7565	490 (6.5)
Female	8001	770 (9.6)	7231	730 (10.1)
Parity				
First birth	9028	928 (10.3)	8100	748 (9.2)
Second birth	5686	618 (10.9)	5068	349 (6.9)
More than third birth	1889	232 (12.3)	1657	127 (7.7)
Birth year				
1997-2001	5959	568 (9.5)	5391	425 (7.9)
2002-2005	4630	539 (11.6)	4091	356 (8.7)
2006-2010	6026	672 (11.2)	5354	444 (8.3)
**Census regions level variables**				
Proportion of professional workers				
Q1 (13.0-33.8%) (affluent)	5453	514 (9.4)	4939	342 (6.9)
Q2 (10.0-13.0%) (middle)	5524	543 (9.8)	4981	441 (8.9)
Q3 (0–10.0%) (poor)	5628	720 (12.8)	4908	442 (9.0)
Proportion of production or transportation workers				
Q1 (0–37.9%) (affluent)	5406	486 (9.0)	4920	374 (7.6)
Q2 (37.9-45.2%) (middle)	5589	575 (10.3)	5014	414 (8.3)
Q3 (45.2-86.3%) (poor)	5610	716 (12.8)	4894	437 (8.9)

†Proportions of PTBs were calculated by dividing the number of PTBs by total births in the corresponding category. Proportions of LBW births were calculated by dividing the number of term LBW by total term births in the corresponding category.

PTB; preterm birth, LBW; low birth weight.

In Table 2, the number and percentage of individual/area-level SEP and personal characteristics separated by proximity to major roads are shown. Mothers whose household occupation was professional and mothers who lived in affluent areas had a tendency to live further from major roads. The correlation between individual and area-level SEP is shown in Table 3, and indicates that mothers whose household occupation was professional tended to live in affluent areas.

**Table 2:** Number and percentages of individual/area-level SEP and personal characteristics by proximity to major roads (≤200 m; >200 m) (n = 16,615)

	**Proximity to major roads**	
	**≤200 m**	**>200 m**
	**n (%)**	**n (%)**
**Individual SEP or personal characteristics**		
Household occupation		
Professional workers (n = 1854)	76 (4.1)	1778 (95.9)
Employees (n = 13070)	767 (5.9)	12303 (94.1)
Self-employed/Part-time workers/Unemployed (n = 952)	57 (6)	895 (94)
Maternal smoking		
Never smoked (n = 15736)	897 (5.7)	14839 (94.3)
Ex- or current-smoker (n = 686)	46 (6.7)	640 (93.3)
Maternal alcohol drinking		
Non-drinker (n = 15674)	902 (5.8)	14772 (94.2)
Drinker (n = 757)	42 (5.5)	715 (94.5)
Paternal smoking		
Never smoked (n = 8650)	477 (5.5)	8173 (94.5)
Ex or current-smoker (n = 7152)	436 (6.1)	6716 (93.9)
Maternal obesity		
Not obese (n = 15215)	855 (5.6)	14360 (94.4)
Obese (n = 1348)	91 (6.8)	1257 (93.2)
Maternal diabetes		
No diabetes (n = 15983)	913 (5.7)	15070 (94.3)
Diabetes (n = 617)	40 (6.5)	577 (93.5)
Maternal hypertension		
No hypertension (n = 15707)	908 (5.8)	14799 (94.2)
Hypertension (n = 893)	45 (5)	848 (95)
**Area-level SEP at census regions**		
Proportion of professional workers		
Q1 (affluent) (n = 5453)	182 (3.3)	5271 (96.7)
Q2 (middle) (n = 5524)	401 (7.3)	5123 (92.7)
Q3 (poor) (n = 5628)	370 (6.6)	5258 (93.4)
Proportion of production or transportation workers		
Q1 (affluent) (n = 5406)	84 (1.6)	5322 (98.4)
Q2 (middle) (n = 5589)	452 (8.1)	5137 (91.9)
Q3 (poor) (n = 5610)	417 (7.4)	5193 (92.6)

SEP; socio-economic position.

**Table 3:** Individual-level SEP within area-level SEP variables (n = 16,615)

	**Area-level SEP at census regions**		
	**Q1 (affluent)**	**Q2 (middle)**	**Q3 (poor)**
	**Proportion of professional workers**		
Household occupation			
Professional workers (n = 1853)	850 (45.9)	545 (29.4)	458 (24.7)
Employees (n = 13062)	4103 (31.4)	4414 (33.8)	4545 (34.8)
Self-employed/Part-time workers/Unemployed (n = 951)	290 (30.5)	317 (33.3)	344 (36.2)
	**Proportion of production or transportation workers**		
Household occupation			
Professional workers (n = 1853)	811 (43.8)	564 (30.4)	478 (25.8)
Employees (n = 13062)	4032 (30.9)	4473 (34.2)	4557 (34.9)
Self-employed/Part-time workers/Unemployed (n = 951)	355 (37.3)	317 (33.3)	279 (29.3)

SEP; socio-economic position.

Table 4 shows the adjusted ORs for the associations of proximity to major roads with preterm birth and term LBW. Although we adjusted for individual SEP (Model 3) and for both individual/area-level SEP variables simultaneously (Model 4), this did not substantially change the point estimates. When we adjusted for both individual/area-level SEP variables simultaneously (Model 4), we found positive associations between proximity to major roads and preterm birth and term LBW. Specifically, living within 200 m of a major road increased the risk of preterm birth by 1.5 times (95% CI: 1.3-1.9) and LBW by 1.2 times (95% CI: 0.9-1.6). Dose–response relationships between proximity to major roads and adverse outcomes were observed when we used the trichotomized exposure indictor.

**Table 4:** Odds ratios (ORs) and their 95% confidence intervals (CIs) between proximity to major roads and preterm births (PTB) or term low birth weight (LBW)

	**Model 1**	**Model 2‡**	**Model 3§**	Model 4**
	**Crude model**	**Adjusted model**	**Individual SEP adjusted**	**Individual/area-level SEP adjusted**
	**OR (95% CI)**	**OR (95% CI)**	**OR (95% CI)**	**OR (95% CI)**
PTB*				
Proximity to major roads				
>200 m	1	1	1	1
≤200 m	1.6 (1.3, 1.9)	1.6 (1.4, 2.0)	1.6 (1.3, 1.9)	1.5 (1.3, 1.9)
Proximity to major roads				
>200 m	1	1	1	1
50-200 m	1.5 (1.3, 1.9)	1.6 (1.3, 2.0)	1.6 (1.3, 1.9)	1.5 (1.2, 1.8)
≤50 m	2.0 (1.2, 3.1)	1.9 (1.2, 3.1)	1.8 (1.1, 3.0)	1.7 (1.0, 2.9)
**Term LBW†**				
Proximity to major roads				
>200 m	1	1	1	1
≤200 m	1.2 (1.0, 1.6)	1.2 (0.9, 1.5)	1.2 (1.0, 1.6)	1.2 (0.9, 1.6)
Proximity to major roads				
>200 m	1	1	1	1
50-200 m	1.2 (1.0, 1.6)	1.2 (0.9, 1.5)	1.2 (0.9, 1.6)	1.2 (0.9, 1.6)
≤50 m	1.3 (0.7, 2.5)	1.4 (0.7, 2.8)	1.5 (0.8, 3.1)	1.5 (0.7, 3.0)

*Subjects were all newborns (n = 16,615).

†Subjects were newborns at term birth (n = 14,836).

‡Adjusted for maternal age, newborn’s sex, maternal alcohol consumption, maternal smoking, maternal body mass index, and paternal smoking. Gestational age was also adjusted in the analysis for term LBW.

§ Additionally adjusted for household occupation.

**Additionally adjusted for the proportion of professional workers in the corresponding census.

PTB; preterm birth, LBW; low birth weight, OR; odds ratio, CI; confidence interval, SEP; socio-economic position.

Although none of the additive interaction terms between air pollution and individual/area-level SEP were statistically significant, some tendencies were observed in the stratified analyses by individual/area-level SEP (Table 5). Mothers with lower individual SEP, i.e., household occupation “Self-employed/Part-time workers/Unemployed”, experienced higher ORs for the association between proximity and term LBW (OR = 3.1, 95% CI: 1.2-8.2) compared with other household occupation categories. In contrast, in the analyses stratified by area-level SEP, mothers who lived in the highest area-level SEP region (i.e., affluent areas) showed slightly higher point estimates for the associations between proximity and adverse birth outcomes compared with those who lived in middle or poor areas. For example, the OR in affluent areas defined by proportion of production or transportation workers was 2.5 (95% CI: 1.1-5.5) for term LBW.

**Table 5:** Odds ratios (ORs) of adverse birth outcomes associated with proximity to major roads (≤200 m) stratified by individual/area-level SEP and parental characteristics

	PTB*		Term LBW*	
	**OR (95% CI)**	**AP (95% CI)**	**OR (95% CI)**	**AP (95% CI)**
**Individual SEP or personal characteristics**				
Household occupation†				
Professional workers	1.6 (0.8, 3.2)	-	1.4 (0.6, 3.3)	-
Employees	1.5 (1.2, 1.9)	0 (−0.6, 0.6)	1.1 (0.9, 1.5)	−0.2 (−1.2, 0.7)
Self-employed/Part-time workers/Unemployed	1.6 (0.7, 3.5)	0.2 (−0.6, 1.0)	3.1 (1.2, 8.2)	0.4 (−0.3, 1.1)
Maternal smoking†				
Never smoked	1.6 (1.3, 2.0)	-	1.2 (0.9, 1.5)	-
Ex- or current-smoker	0.8 (0.3, 1.8)	−0.5 (−1.6, 0.7)	2.4 (1.0, 6.2)	0.5 (0, 1.0)
Maternal alcohol drinking†				
Non-drinker	1.6 (1.3, 1.9)	-	1.2 (1.0, 1.6)	-
Drinker	1.2 (0.4, 3.3)	−0.2 (−1.3, 1.0)	0.7 (0.1, 3.3)	−0.8 (−3.6, 1.9)
Paternal smoking†				
Never smoked	1.7 (1.3, 2.2)	-	1.2 (0.8, 1.7)	-
Ex or current-smoker	1.4 (1.1, 1.9)	−0.1 (−0.6, 0.3)	1.2 (0.9, 1.8)	−0.1 (−0.9, 0.8)
Maternal obesity†				
Not obese	1.6 (1.3, 2.0)	-	1.2 (0.9, 1.6)	-
Obese	1.3 (0.7, 2.5)	−0.1 (−0.8, 0.6)	1.6 (0.7, 3.6)	0.2 (−0.5, 0.9)
Maternal diabetes†				
No diabetes	1.5 (1.2, 1.8)	-	1.2 (0.9, 1.6)	-
Diabetes	4.4 (1.9, 10.0)	0.6 (0.4, 0.9)	1.4 (0.3, 7.0)	0 (−1.5, 1.5)
Maternal hypertension†				
No hypertension	1.5 (1.2, 1.8)	-	1.3 (1.0, 1.6)	-
Hypertension	2.6 (1.2, 5.4)	0.4 (0, 0.9)	0.5 (0.1, 2.5)	−1.1 (−4.2, 2.0)
**Area-level SEP at census regions**				
Proportion of professional workers§				
Q1 (affluent)	1.9 (1.2, 3.0)	-	1.4 (0.8, 2.6)	-
Q2 (middle)	1.6 (1.2, 2.2)	−0.2 (−0.8, 0.4)	1.3 (0.9, 1.9)	0 (−0.6, 0.6)
Q3 (poor)	1.4 (1.1, 1.9)	−0.2 (−0.7, 0.4)	1.1 (0.7, 1.6)	−0.2 (−1.0, 0.6)
Proportion of production or transportation workers§				
Q1 (affluent)	2.5 (1.4, 4.4)	-	2.5 (1.1, 5.5)	-
Q2 (middle)	1.2 (0.9, 1.7)	−0.9 (−2.1, 0.2)	1.2 (0.8, 1.8)	−0.9 (−2.5, 0.7)
Q3 (poor)	1.6 (1.2, 2.2)	−0.3 (−0.9, 0.4)	1.0 (0.7, 1.6)	−1.1 (−2.9, 0.7)

ORs were estimated against subjects living in the area more than 200 m from major roads. Proportions attributable to the interaction (AP) between the respective factors and proximity to major roads are also shown.

*Subjects were all newborns (n = 16,615) in the analyses for PTB and subjects were newborns at term birth (n = 14,836) in the analyses for term LBW.

†Adjusted for maternal age, newborn’s sex, maternal smoking, maternal alcohol consumption, maternal body mass index, paternal smoking, household occupation, and the proportion of professional workers in the corresponding census. Each confounder was excluded when subjects were stratified by the corresponding variable. Gestational age was also adjusted in the analysis for term LBW.

§Adjusted for maternal age, newborn’s sex, maternal smoking, maternal alcohol consumption, maternal body mass index, paternal smoking, and household occupation. Gestational age was also adjusted in the analysis for term LBW.

AP; Proportion attributable to the interaction, CI; confidence interval, LBW; low birth weight, OR; odds ratio, PTB; preterm birth, SEP; socio-economic position.

Regarding stratified analyses by parental characteristics, mothers who reported a past history of diabetes and hypertension experienced a higher OR for the association between proximity and preterm birth (Table 5): e.g., OR = 4.4 (95% CI: 1.9-10) for those with diabetes and OR = 1.5 for those without diabetes (95% CI: 1.2-1.8), and the corresponding AP was 0.6. Thus, it could be argued that 60% of preterm births among those with both exposure conditions (living within 200 m from major roads and diabetes) were attributable to their interaction. In contrast, ex- or currently smoking mothers experienced a higher OR for the association between proximity and term LBW.

## Discussion

In this study, we evaluated how SEP at both individual and group/area-levels and parental characteristics modified the relationship between air pollution and adverse birth outcomes (preterm birth and term LBW), using proximity to major roads as an exposure indicator. We observed that mothers from lower individual SEP, defined by household occupation, had higher effect estimates for term LBW compared with mothers with higher individual SEP. In contrast, mothers from higher area-level SEP regions tended to have higher effect estimates for both preterm birth and term LBW compared with mothers from lower area-level SEP regions. In addition, maternal diabetic, hypertensive, and smoking status modified the association between proximity and birth outcomes.

There is a lack of studies on this topic from Asian countries. Extrapolation from studies conducted in different areas may be inappropriate because of differences in pollution characteristics, health care systems, meaning of SEP indicators, study designs, and demographics of the population.

The findings of this study are consistent with recent studies suggesting that air pollution exposure increases the risk of preterm birth and term LBW [6,7]. Our study additionally supports the finding that lower individual or area-level SEP is associated with adverse birth outcomes [36,37], even in an egalitarian country like Japan [38].

In the analyses stratified by SEP, although not statistically significant, mothers with lower individual SEP showed susceptibility to air pollution as expected, especially in the “Self-employed/Part-time workers/Unemployed” occupational category. This finding is consistent with previous air pollution studies examining cardiopulmonary outcomes among the adult population. These studies showed susceptibility associated with lower education or income at the individual level [18-21]. In contrast, mothers who lived in higher area-level SEP regions tended to have greater effect estimates for adverse birth outcomes stemming from air pollution exposure. This finding is similar to the study in Canada, which showed stronger effects in higher area-level SEP neighborhoods [22], and contradicts some previous studies showing increased susceptibility in lower SEP areas [23-25].

Moreover, we found that maternal diabetic and hypertensive status modified the relationship between air pollution and preterm birth. This finding is consistent with previous air pollution studies in adult settings, which showed that air pollution-related health effects were stronger among diabetic or hypertensive patients [9,10,39]. Indeed, recent reviews suggest that the potential mechanism for the association between air pollution and adverse birth outcomes is impaired placental function (placental oxygen and nutrient transport), in turn brought about by oxidative stress, inflammation, coagulation, impaired endothelial function, and hemodynamic responses [3,40]. Diabetes and hypertension can cause preeclampsia, infections, vascular complications, and placental insufficiency during pregnancy [41], which may support the susceptibility of diabetic and hypertensive mothers. The reason why maternal smoking status modified the association between proximity and term LBW may also be related to this mechanism, because smoking is associated with placental insufficiency during pregnancy [41].

Although most air pollution studies used register-based participants [22-24,42,43], our study participants were maternal-newborn pairs who attended one general hospital with a perinatal center. Not all babies in the western part of Shizuoka are born in this particular hospital and this hospital-based sampling method may introduce selection bias. However, the proportion of preterm births delivered in this hospital was higher mainly among mothers residing far from the hospital. The proportion of preterm births was 7.4% among mothers residing close to the hospital (lowest 10th percentile of the distance from the hospital), and 23.4% among mothers residing far from the hospital (highest 10th percentile of the distance). The hospital is located relatively close to major roads [8], hence this type of selection bias, if it exists, would likely only underestimate the results.

In the present study, we used mothers’ proximity to major roads as an index for air pollution exposure, since air-monitoring stations are limited in their coverage and spatial resolution. However, there is a possibility that proximity to major roads is limited in temporal resolution. Traffic information in this study was obtained from the Road Traffic Census conducted in 2005, and traffic volume data were recorded for only 1 day, during the period from September to November in 2005. Thus, our exposure indicator does not reflect year-to-year or seasonal variations in traffic exposure. Because major roads did not change, the traffic volume would not have varied substantially in the year-to-year comparison. The average changes (increase) in daily traffic volume over a 12-hour time period for the whole Shizuoka prefecture were 135 vehicles from 1999 to 2005 and 200 vehicles from 2005 to 2010, respectively [44]. However, our exposure indicator did not provide any indication of seasonal variation.

Exposure misclassification could have occurred because of mothers’ movement during pregnancy, as we used maternal residential information at delivery. Therefore, we restricted subjects to those who lived in the western part of Shizuoka prefecture to reduce the possibility of misclassification. Although we could not obtain information about maternal movement, such possible non-differential exposure misclassification between the two categories (≤200 m; > 200 m) would likely have moved effect estimates toward the null [45].

In the present study, we used household occupation as our measure of individual SEP. However, the category was relatively crude. Moreover, we had to combine three categories (Self-employed; Part-time workers; Unemployed) because of limited numbers. Furthermore, we did not have other individual SEP information such as education, income, or wealth [46]. These issues might have affected the observed non-significant interactions between air pollution and adverse birth outcomes in the present study. Additionally, we could not separate mothers who quit smoking before pregnancy (ex-smoker) from mothers who quit smoking during pregnancy, which may have been a potentially confounding factor in the present study.

## Conclusions

The present study demonstrated that air pollution is an independent risk factor for adverse birth outcomes, and lower SEP at both individual and area-levels was associated with the increased occurrence of adverse birth outcomes. Although the interaction was not statistically significant, mothers with lower individual SEP and mothers living in higher SEP regions tended to be susceptible to the adverse effect of air pollution. Moreover, maternal diabetic, hypertensive, and smoking status increased susceptibility to the effect. Further studies are needed to identify mothers vulnerable to air pollution to reduce reproductive adverse outcomes of newborns.

## Consent

According to the ethical guidelines for epidemiological research in Japan, no informed consent from participants was needed because data were existing materials and analyzed anonymously.

## Abbreviations

AP: Proportion attributable to the interaction; BMI: Body mass index; CI: Confidence interval; GIS: Geographic Information System; LBW: Low birth weight; OR: Odds ratio; SEP: Socio-economic position.

## Competing interests

The authors declare that they have no competing interests.

## Authors’ contributions

Study concept and design: TY, SK, ST, HD, IK; Data collection: HN, TM; Data handling: TY, SK; Analysis: TY; Interpretation of data: TY, SK, ST, HD, IK; Drafting of the manuscript: TY, IK; Critical revision of the manuscript: HN, SK, ST, TM, HD; Study supervision: IK. All authors read and approved the final manuscript.
